# Highlights of the 30th Annual Congress of the EANM, Vienna 2017: “Yes we can – make nuclear medicine great again”

**DOI:** 10.1007/s00259-018-4029-9

**Published:** 2018-05-03

**Authors:** Stefano Fanti, Rachele Bonfiglioli, Clemens Decristoforo

**Affiliations:** 1 Istituto di Medicina Nucleare, Dipartimento di Medicina Specialistica, Diagnostica e Sperimentale, Alma Mater Studiorum Università di Bologna, Bologna, Italy; 2 Department of Nuclear Medicine, Medical University Innsbruck, Anichstrasse 35, A-6020 Innsbruck, Austria

**Keywords:** EANM 2017, Annual congress, Abstracts, Highlights, Nuclear medicine, Radionuclide therapy and dosimetry, Radiopharmaceuticals, PET, SPECT

## Abstract

The 30th Annual Congress of the European Association of Nuclear Medicine (EANM) was held in Vienna, Austria, from 21 to 25 October 2017 under the chairmanship of Professor Francesco Giammarile. As always, the Congress was a great success: more than 6,379 participants came from 90 countries from all continents. Participants were presented with an excellent programme consisting of symposia, and scientific and featured sessions, CME sessions, and plenary lectures. These lectures were devoted to nuclear medicine imaging and therapy, including hybrid imaging and molecular life sciences. Additionally, the latest technology and innovations in the field were presented, and added to the success of the Congress. This review summarizes the major scientific contributions which were selected from more than 1,900 submitted abstracts, and presented in the closing highlights session. They cover the diverse areas of nuclear medicine, with particular focus on oncology, cardiovascular science, neurology, technological innovation and novel tracers, and also other clinical sciences. A particular focus of the Congress was on targeted radionuclide-based therapies, which all show promising and great innovations. The Congress was a unique opportunity to be thoroughly updated on this research. This Highlights Lecture could only be a brief summary of the large amount of data presented and discussed during the meeting, which can be found in much greater detail in the Congress proceedings book, published as volume 44, supplement 2 of the *European Journal of Nuclear Medicine and Molecular Imaging* in October 2017.

## Introduction

From 21 to 25 October 2017 the 30th Annual Congress of the European Association of Nuclear Medicine (EANM 2017) took place in Vienna, Austria. With more than 6,379 participants on site and 1,571 online this congress is by far the most important European event in nuclear medicine, bringing together a multidisciplinary community involved in the different fields of nuclear medicine. Participants from 90 different countries attended, with more than 1,300 attendees from non-European countries, indicating the worldwide importance of the meeting. The Scientific Programme Committee chaired by Francesco Giammarile organized the programme that covered all topics relevant to nuclear medicine comprising 529 oral and 1,021 poster presentations in more than 100 sessions during the meeting.

**Fig. 1:**
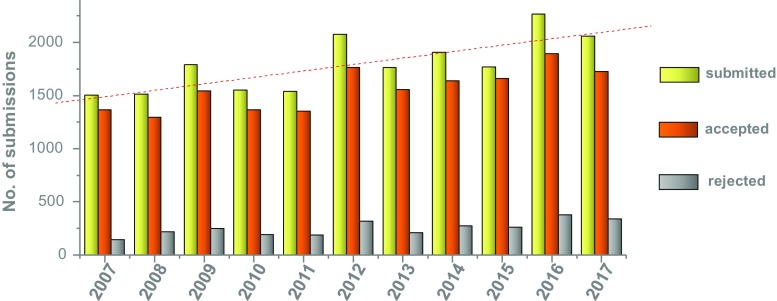
Numbers of abstracts submitted, accepted and rejected for presentation at EANM annual meetings since 2007

**Fig. 2:**
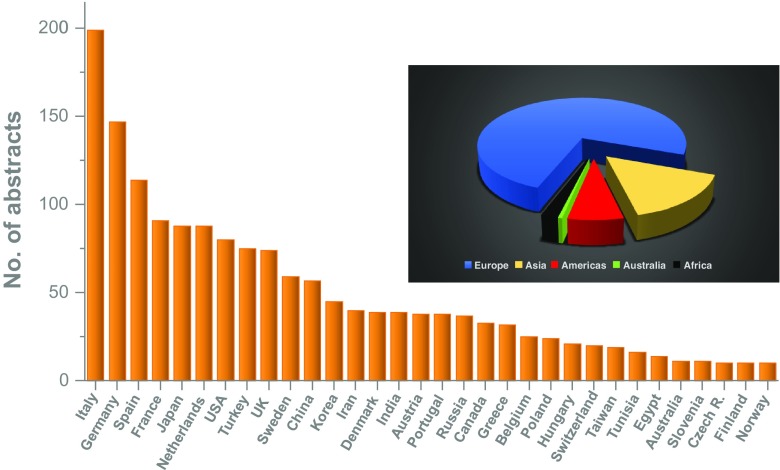
Abstracts submitted to EANM 2017 by country and continent

Overall 1,925 scientific abstracts were submitted and finally 1,613 were accepted (16.2% rejection rate). This number was only slightly topped by last year’s meeting in Barcelona and the 25th Annual Meeting in Milan 2012 (see Fig. 1). The highest numbers of abstracts were received from Italy (12.3%), Germany (9.1%), Spain (7.1%) and France (5.6%). At 5.5%, Japan was the best-represented non-European country. Overall 75% of abstracts were from Europe, 15% from Asia, 7% from the Americas, and a small fraction from Africa and Australia; details are provided in Fig. 2. Looking at the cities from where abstracts were submitted, most European abstracts came from Munich (321), followed by Rome (265), London (239) and Genova (237). Most abstracts from outside Europe came from Tehran (141), Seoul (131), Beijing (126) and New York (110).

The abstracts covered a wide range of topics. Even though research related to applications of nuclear medicine in oncology dominated, an equal number of abstracts related to other clinical areas including cardiovascular science (8.1%), neurosciences (8.8%) and basic sciences including physics and instrumentation (12.9%) and radiopharmacy/radiochemistry (11.0%). The translational and multidisciplinary character of the Congress was indicated by the fact that the specific topics Translation (‘from molecule to man’, M2M) included 400 abstracts and Dosimetry & Radionuclide Therapy (DoMoRe) included 457 abstracts.

However, it is not the overall numbers that are important, but the content of the abstracts regarding novel findings and their impact on advances in the field of nuclear medicine. The EANM 2017 Highlights Lecture included a selection of the work presented at the meeting. The abstracts were initially rated by selected experts in the individual categories, who also proposed 421 of the best abstracts for possible inclusion in this year’s Highlights Lecture. From these abstracts, 46 were finally chosen for inclusion as examples of a large number of submissions that were often comparable and scientifically of equal importance. The authors (S.F., C.D.) had the honour to present the Highlights Lecture at this meeting. In preparing for the task of selecting the submissions to be included, they asked: Could this congress “make nuclear medicine great again”? (a reference to the recent campaign of the US president).

We invite readers to judge for themselves and to read this year’s highlights of EANM 2017 in different categories. As there is no Oscar statue awarded, we proposed that the submitting scientists be honoured with a virtual George and Marie statue, in memory of George von Hevesy and Marie Curie-Sklodowska, considered the pioneers of diagnostic and therapeutic nuclear medicine applications, who, we are sure, would have enjoyed the presentations would have acknowledged their scientific quality.

## Physics and instrumentation

Novel instrumentation for improved diagnostic procedures has been a major driving force for advancing molecular imaging, in particular for PET applications over recent years, accompanied by applied physics to realize the full potential of the new technologies. This meeting continued this trend and gave a glimpse of what may lie available in years to come.

Cates and Levin [1] (Stanford, USA) presented an outstanding paper on a promising new PET detector design. They changed the readout of the detectors from the end to the side which led to lower losses in signal while also allowing a much higher timer resolution down to 100 ps. Implemented in a clinical detector system an increase in signal to noise ratio by a factor of 5 could be achieved. These innovations could be packaged into a practical clinical detector module with mixed analogue-digital multiplexing schemes that require a low number of readout channels, leading to a faster and more sensitive PET detector.

**Fig. 3:**
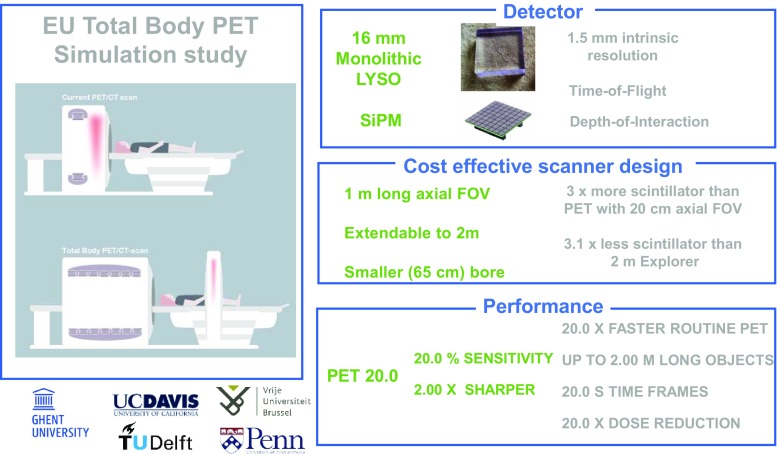
PET 20.0: a cost-efficient, 2.00-mm resolution total body monolithic PET system with very high sensitivity and an adaptive axial field of view up to 2.00 m, presented by Vandenberghe et al. of the University of Ghent in collaboration with other Dutch, Belgian and US institutions [2]

A study in collaboration between Belgian, Dutch and US centres was presented by Vandenberghe et al. [2]. They presented a cost-efficient 2-mm resolution whole-body PET design with a 1 m field of view extendable to 2 m, also based on a cost-effective design. With this approach they were able to achieve a 20 times faster PET acquisition or alternatively a 20-fold reduction in dose, further improving and simplifying PET scans (Fig. 3).

Novel technologies also have to be translated into clinical practice and show their added value in a larger setting. In this respect two presentations reflect such developments. One important topic is to harmonize readouts to establish clinical standards in particular for the quantification of molecular processes, which is one of the advantages of nuclear imaging methods. Kaalep et al. [3] (Tallinn, Estonia) presented a collaborative study (several European and one US centre) investigating the feasibility of harmonizing the performance of state-of-the-art PET/CT systems. They performed phantom studies on the newest time of flight (ToF) PET/CT systems available with resolution modelling/point spread function (PSF) technologies, and showed that it is possible to harmonize the systems’ retard, which may provide the basis for a wider study of system harmonization.

Novel tools to utilize the full data generated by novel imaging studies are advancing rapidly. Grueneisen et al. [4] (Essen, Germany) used radiomics to predict the N and M stage of cervical cancer using PET/MRI. They used the PET/MRI signal and both PET and MRI images and applied a radiomics approach to define certain features typical of certain types of tumour. They achieved very high sensitivity and specificity for predicting N and M stage of cervical cancers, providing a number of noninvasive biomarkers and facilitating improved tumour evaluation and treatment planning. Thus, they were also able to show the benefit of PET/MRI over MRI alone.

Novel technologies also have to show benefits in terms of patient safety. In this respect the presentation of Petoussi-Henss et al. [5] (Neuherberg, Germany) should be mentioned. These authors described a voxel-based dosimetry program able to accommodate the new dosimetric International Commission on Radiological Protection (ICRP) framework for simplified implementation in the clinical setting to provide more accurate estimation of radiation dose in individual patients.

## Cardiovascular science

In this category a large number of submitted abstracts were on the subject of the diagnosis of myocardial infarction. Even though the topic as such is not novel, new opportunities in this area are emerging, in particular for imaging plaques and thrombosis. Another novel topic, which was dealt with in several presentations, is PET imaging of cardiac amyloidosis.

Rischpler et al. [6] (Munich, Germany) evaluated the relationships among myocardial inflammation, the area at risk (AAR), oedema and tissue damage using different approaches. In particular, they compared data derived from ^99m^Tc-sestamibi SPECT and FDG PET/MRI and other MRI information. They found that the AAR was quite similar between MRI and sestamibi SPECT, but at the same time there was a poor correlation between oedema and AAR, and the association between oedema and irreversible tissue damage and inflammation indicates that MRI should not be used for AAR assessment.

Novel targets for cardiovascular applications were also presented. A very innovative exploratory study involving collaboration between the two universities of Munich and a Belgian centre was presented by Varasteh et al. [7]. They targeted the mannose receptor (CD206) expressed on activated reparative macrophages with a ^68^Ga-NOTA anti-CD206 Nanobody for imaging the mannose receptor in mice with experimental myocardial infarction. With this approach they were able to depict the area where activated macrophages accumulate, which is strictly associated with the infarction healing process. In a second presentation by the same group [8], the use of the same radiotracer (^68^Ga-NOTA anti-CD206 Nanobody) for a different application, to identify atherosclerotic plaque in apolipoprotein E-knockout (ApoEKO) mice, was reported, suggesting that NOTA anti-CD206 Nanobody is also a promising tracer for non-invasive detection of atherosclerotic plaques.

**Fig. 4:**
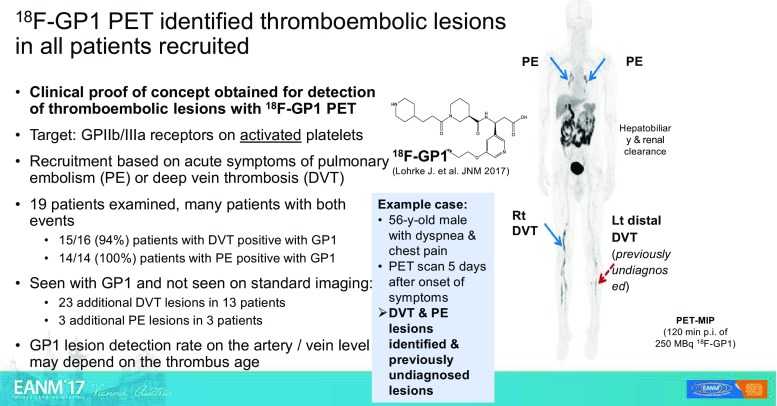
Diagnosis of deep venous thrombosis and pulmonary embolism using ^18^F-GP1 positron emission tomography. An exploratory open-label study from Seoul [9] showing excellent targeting of GPIIb/IIIa receptors on activated platelets leading to high sensitivity and specificity

An exploratory open-label clinical study was presented by Kim et al. [9] (Seoul, Korea). They reported that it may be possible to diagnose deep venous thrombosis and pulmonary embolism using ^18^F-GP1 PET/CT (Fig. 4). They targeted a glycol-protein receptor (GPIIb/IIIa) overexpressed on activated platelets and they were able to show the presence in vivo of pulmonary embolism as well as deep venous thrombosis with an extremely high sensitivity in the range 94–100%. They were also able to detect additional sites of venous thrombosis that were not detected by conventional imaging methods.

A representative study of cardiac amyloidosis imaging is that of Genovesi et al. [10] (Pisa, Italy) who investigated the use of ^18^F-florbetaben PET/CT in this indication. They used dynamic scans and were able to clearly discriminate the patterns of accumulation in the two different types of cardiac amyloidosis, transthyretin-related amyloidosis (ATTR) and light chain-related amyloidosis (AL). On the basis of the different wash-in and wash-out characteristics of the tracer in the ATTR and AL groups, ^18^F-florbetaben PET/CT was able to differentiate the two types of amyloidosis indicating that brain involvement in the context of systemic amyloidosis in ATTR patients could not be due to the same type of amyloid deposits, which involve other organs including the heart and bone marrow.

## Basic science and preclinical developments

Nuclear medicine has the unique potential to become great because of translation of developments in basic biomedical sciences into clinical applications. A number of presentations exemplify this in an impressive way with novel radiotracers and basic studies on novel, clinically relevant targets.

**Fig. 5:**
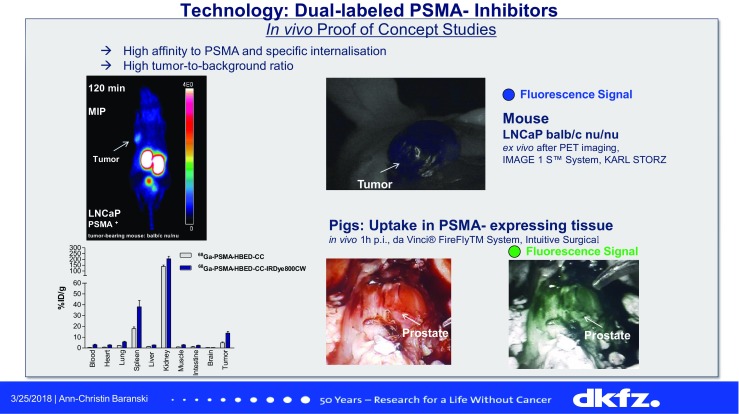
^68^Ga-PSMA-HBED-CC-IRdye800CW, a novel dual labelled PSMA inhibitor with promising properties that may allow PET/CT to be combined with fluorescence-guided surgery for the evaluation of prostate cancer [12]

Amor-Coarasa et al. [11] (New York City, USA) reported on the development of a new ligand, ^18^F-RPS-544, that targets the CXCR4 receptor, and is relevant for the evaluation of tumour progression and metastatic potential. They performed PET studies in nude mice bearing bilateral (CXCR4+ and CXCR4−) PC3 xenograft tumours and showed excellent tumour targeting, a sevenfold improvement over existing ^18^F-fluorinated ligands reported to date resulting in high tumour to background ratios. Baranski et al. [12] (Heidelberg, Germany) presented a study on a novel dual labelled compound based on PSMA-11 (Fig. 5) evaluating the potential of a combination of preoperative staging by PET/CT and fluorescence-guided surgery for detecting metastasis or neoplastic lymph nodes in patients with prostate cancer. They showed that this compound (^68^Ga-PSMA-HBED-CC-IRdye800CW) provides PSMA-specific PET and fluorescence signals with promising pharmacokinetic properties in the surgical setting indicating a high potential for future clinical translation.

Heskamp et al. [13] (Nijmegen, The Netherlands) presented a study exploring the potential of PD-L1 microSPECT/CT imaging for monitoring changes in PD-L1 expression in tumours during radiotherapy (RT), of relevance in patients receiving immune checkpoint inhibitor treatment. They analysed an animal model which was irradiated and 1 day later received 25 MBq ^111^In-labelled anti-PD-L1 antibody followed by microSPECT/CT imaging, in comparison with non-irradiated control mice. They showed that expression of the PD-L1 target increases after irradiation. Xu et al. [14] (Beijing, China) presented a similar study. These authors used an anti-PD-L1-labelled antibody, ^64^Cu-NOTA-Ab1881, in an animal model expressing the PD-L1 target. Using this labelled antibody they were able to predict therapy outcome, distinguishing PD-L1-positive and PD-L1-negative xenografts in the mouse model with PET imaging, and also demonstrated therapeutic efficacy, which was highly correlated with the tumour uptake of ^64^Cu-NOTA-Ab1881. These studies suggested that targeting PD-L1 could be used to monitor PDL1 expression during immune checkpoint inhibitor administration to select patients benefiting from treatment and to develop optimal treatment strategies.

D’Alessandria et al. [15] (Munich, Germany) presented the results of a study comparing galectin-3 immunotargeting and radioiodine imaging in thyroid orthotopic tumour models. These authors showed that galectin-3 is expressed in thyroid carcinomas but is not related to the sodium iodide symporter in animals models. This approach could allow evaluation of thyroid cancer independently from the sodium iodide transporter status, and is a promising approach to changing the management of patients with thyroid cancer who lose radioiodine uptake in the clinical setting.

In relation to radionuclide therapy, a presentation by Altai et al. [16] (Uppsala, Sweden) focused on pretargeted radionuclide therapy. They used a HER-2-targeting Affibody modified with peptide nucleic acids (PNAs) that allowed pretargeting by first injecting the PNA-Affibody followed by ^177^Lu-PNA in the therapeutic setting. The use of Affibody-based PNA-mediated pretargeting was able to deliver high doses to tumours with a very good therapeutic effect in a preclinical model, while sparing the kidneys, which has so far been the problem with this therapeutic approach.

**Fig. 6:**
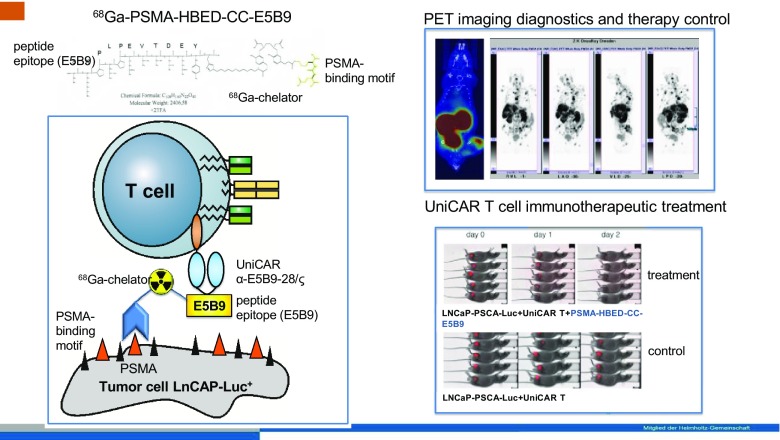
A theranostic approach, presented by Bergmann et al. [17], used to monitor a novel immunotherapeutic technique (UniCAR T) based on site-directed target molecules that upon binding selectively turn on the immunotherapeutic action of T cells. A ^68^Ga-PSMA targeting molecule is able to initialize the therapeutic effect and at the same time allows the targeting efficiency to be monitored by PET

With a special kind of theranostic approach, Bergmann et al. [17] (Dresden, Germany) addressed a new concept that involved targeting PSMA-expressing tumours using a novel immunotherapeutic technique (UniCAR T). Using a ^68^Ga-labelled modified PSMA 11 molecule with a recognition site for T cells (HBED-CC-PSMA-E5B9, PSMA-TM), the therapeutic efficacy of T cells is switched on leading to site-directed therapy that was demonstrated in an experimental animal model (Fig. 6). PSMA-TM was shown to be a useful PET imaging agent for noninvasively following the progress of individualized PSMA-directed immunotherapeutic tumour treatment. This is a nice proof of concept for the use of nuclear imaging to plan, monitor and optimize patient treatments based on novel immunotherapy concepts.

## Neuroscience

A great variety of presentations covered novel applications in neuroscience, reflecting the high potential of nuclear medicine for visualizing numerous targets for a variety of diseases. In particular, interesting contributions in relation to Parkinson’s disease (PD), cognitive impairment and neuroinflammation were presented.

Hooshyar Yousefi et al. [18] (Munich, Germany) reported a first imaging study investigating a therapeutic target in PD, alpha-synuclein (α-syn), using a fluorinated compound, ^18^F-DABTA. They were able to demonstrate in an animal model a significantly increased PET signal in regions with abundant α-syn (substantia nigra, thalamus, cerebellum and brainstem). This selective α-syn tracer may allow early diagnosis of α-synucleinopathies such as PD and dementia with Lewy bodies.

Another target, glutamate receptor type 5 (mGluR5), is a potentially important regulator of the most common excitatory neurotransmitter system in the brain. Kang et al. [19] (New York, USA) explored the possibility of a correlation between mGluR5 and PD using the selective mGluR5 PET probe ^18^F-FPEB in nine patients with PD compared with a control group, and showed a significant enhancement of binding of the radiotracer in PD patients compared with the healthy controls. This study indicates that the response to dopaminergic denervation in PD is mediated in part by the mGluR5 system.

A very interesting first-in-human study was presented by Arlicot et al. [20] (Tours, France). They demonstrated a very reliable and convenient PET protocol for dopaminergic dysfunction imaging using a novel fluorinated compound, ^18^F-LBT-999, a neuronal dopamine transporter ligand, that could potentially replace current SPECT imaging. ^18^F-LBT-999 PET imaging showed the conventional depiction of the basal ganglia in healthy volunteers, but showed a significant decrease in uptake in patients with PD, demonstrating easy quantification and excellent discrimination between patients with early PD and healthy controls.

**Fig. 7:**
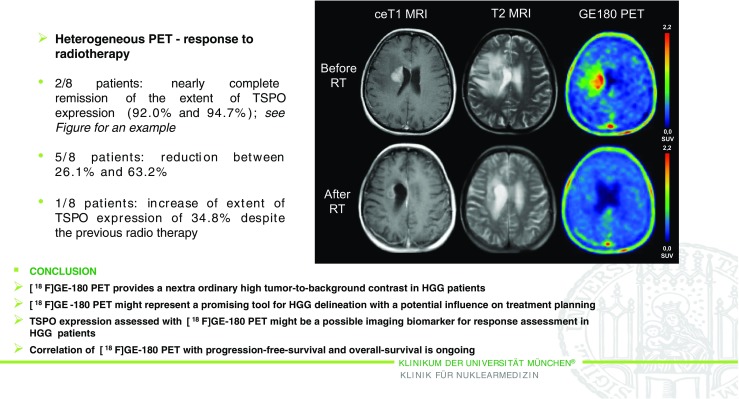
TSPO PET imaging of high-grade glioma (HGG) using the novel ligand ^18^F-GE-180. First-in-human results in the course of radiotherapy [21]

Another highly interesting abstract was presented by Unterrainer et al. [21] (Munich, Germany). They reported a study of the mitochondrial translocator protein (TSPO) expressed in neuroinflammation (Fig. 7) and upregulated in high-grade glioma (HGG), and ^18^F-GE-180, a novel third generation TSPO receptor ligand. In this first-in-human study performed in 12 patients with HGG, ^18^F-GE-180 PET was shown to be a promising tool for assessing the extent of the malignancy before and after radiation therapy, and also for evaluating response to radiation therapy.

Tau radiotracers mimic intracellular tau deposition, a pathological feature of the main neurodegenerative disorders. Seibyl et al. [22] (New Haven, USA) presented a new fluorinated compound, ^18^F-PI-2620. They performed first-in-human PET studies in patients with Alzheimer’s disease (AD) and progressive supranuclear palsy (PSP), in comparison with a healthy group, revealing a different pattern of accumulation in AD patients, especially in the temporal lobes, and different patterns of distribution in PSP patients, demonstrating a high target specificity and high signal in regions of expected tau pathology.

## New radiopharmaceuticals: clinical applications

Based on many excellent submissions describing the first clinical applications of novel radiopharmaceuticals, a separate category was dedicated solely to this topic, involving imaging of beta cells, apoptosis, adrenal and neuroendocrine tumours.

**Fig. 8:**
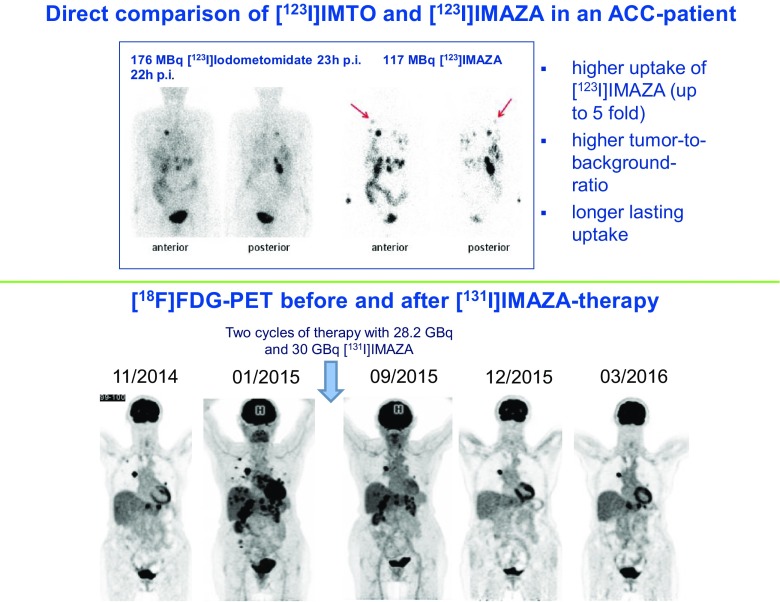
^123/131^I-IMAZA as a new theranostic tool in patients with advanced adrenocortical carcinoma [25]

Boss et al. [23] (Nijmegen, The Netherlands) used ^68^Ga-exendin-4 PET/CT to image beta cells in patients after gastric bypass surgery. Those patients who benefited most from bypass surgery had an increased beta-cell mass (BCM), whereas BCM was lower in patient with incomplete remission. Quantifying BCM could therefore predict the outcome of surgery and could be used for personalizing treatment. Dubash et al. [24] (London, UK) reported a first-in-human study with ^18^F-ICMT-11, targeting caspase 3/7, which is involved in apoptosis, as a novel marker for programmed cell death. They evaluated 17 patients with breast and lung cancer, and analysed the pattern of expression of this caspase. They concluded that fluorinated ICMT-11 can be used as a potential biomarker for chemotherapy-induced apoptosis. Schirbel et al. [25] (Wurzburg, Germany) presented a new compound called IMAZA, labelled with ^123^I and ^131^I, for targeting advanced adrenocortical carcinoma. Compared to the first-generation iodometomidate compound (IMTO), ^123^I-IMAZA had higher metabolic stability resulting in highly improved image contrast, which allowed translation to therapy of this rare tumour with ^131^I-IMAZA with very promising therapeutic efficacy and few side effects (Fig. 8).

Rischpler et al. [26] (Munich, Germany) presented data on LMI1195, a novel ^18^F-labelled radiotracer for noninvasive PET imaging of the norepinephrine transporter, which is expressed in phaeochromocytoma and paraganglioma. LMI1195 demonstrated better imaging properties than ^123^I-MIBG, and is highly promising for the confirmation, exclusion and staging of tumours of the adrenal medulla or the sympathetic trunk with the potential to move from SPECT to PET in this indication. An interesting study was presented by Zheng et al. [27] (Beijing, China). They reported the first-in-human application of a dual-targeted radiopharmaceutical, ^68^Ga-TATE-RGD, that binds to both the somatostatin receptor and integrin αvβ3. In 37 patients with non-small-cell lung cancer and neuroendocrine tumours (NET) they found that ^68^Ga-TATE-RGD provided better quality images than other single-targeted radiopharmaceuticals.

## Clinical oncology

The broad progress in clinical applications in oncology was thoroughly represented in the meeting. A variety of topics, exemplified by an excellent presentation on radiomics, CXCR4 targeting, breast cancer imaging and hypoxia, were covered.

Liu et al. [28] (New York, USA) reported the identification of early small lung cancer with aggressive features (solid and micropapillary patterns) using a combination of PET and CT radiomics analysis. They extensively reviewed 170 radiomics features to identify aggressive components, and found a very interesting correlations between 29 CT and 65 PET radiomics features. Both PET and CT radiomics features were found to be potential biomarkers of aggressive lung adenocarcinoma subtypes with the potential to discriminate between patients requiring rather extensive surgery and those in whom a conservative surgical approach would be sufficient. An interesting study was presented by Watts et al. [29] (Chandigarh, India), who investigated the use of ^68^Ga-pentixafor PET/CT, targeting the chemokine receptor CXCR4, in 25 lung cancer patients. They found that this tracer shows different degrees of uptake in biopsied lung carcinoma depending on the histology, and this result is correlated with CXCR4 expression. This novel PET tracer therefore seems to be useful for imaging and also for stratification, with prognostic implications.

Paquette et al. [30] (Sherbrooke, Canada) reported the use of the combination of ^18^F-FDG PET/CT and oestrogen receptor imaging with ^18^F-4FMFES in patients with breast cancer, in particular in 13 patients with the luminal subtype, which is known to be difficult to assess using FDG alone. The combination of the two tracers overcame the limitation of FDG and achieved a better detection rate and diagnostic confidence, and more accurate staging. Medina Ornelas et al. [31] (Mexico City, Mexico) reported an interesting study of the use of ^68^Ga-PSMA PET/CT in the evaluation of metastatic breast cancer. This tracer is a marker of angiogenesis in many types of solid tumour, and in this particular setting was found to be very accurate with expression of PSMA in metastatic sites observed in 100% of 11 patients. This result could help select patients with tumours with high expression of PSMA for targeted therapy with ^177^Lu-PSMA.

**Fig. 9:**
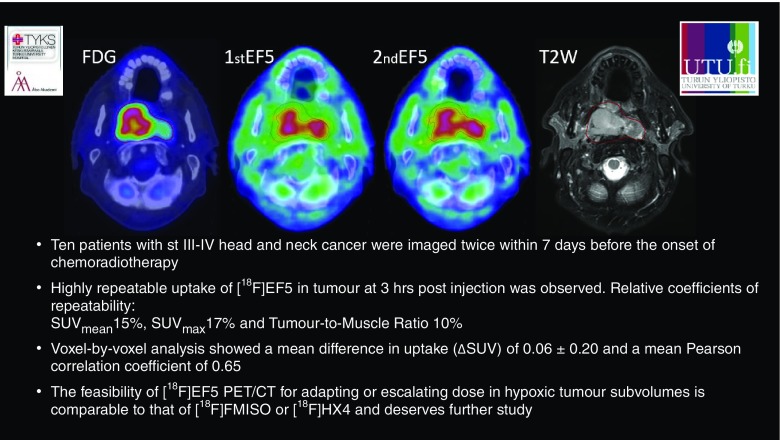
Repeatability of tumour hypoxia imaging using ^18^F-EF5 PET/CT in head and neck cancer [32]

Silvoniemi et al. [32] (Turku, Finland), presented a study that focused on hypoxia imaging, because lack of oxygen contributes to RT resistance and more aggressive behaviour of several types of cancer (Fig. 9). In 11 patients with head and neck cancer, PET/CT with ^18^F-EF5 showed very good repeatability in the imaging of tumour hypoxia, which is very important especially for planning hypoxia-targeted treatment interventions.

## Prostate

Prostate cancer imaging and therapy, driven by the success of PSMA ligands, has been a well-represented topic throughout all EANM meetings over recent years. The high interest and scientific focus is exemplified by a number of excellent presentations dealing with RT planning, use of PET/MRI and novel prostate cancer targeting radiopharmaceuticals.

**Fig. 10:**
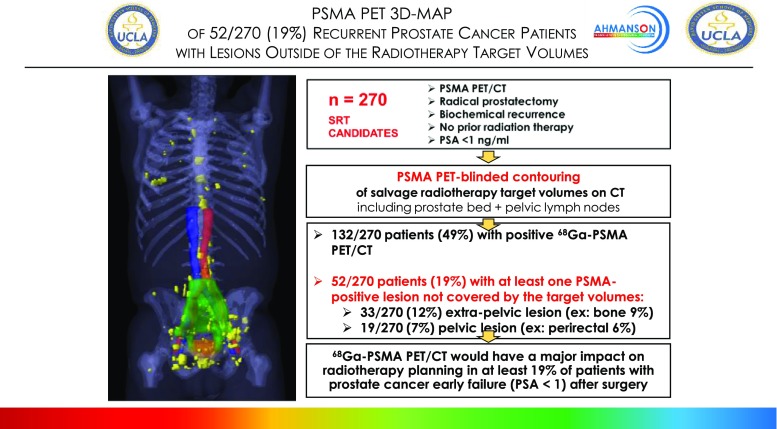
^68^Ga-PSMA PET/CT mapping of early biochemical recurrence after primary surgery in 270 Patients with PSA < 1.0 ng/ml, indicating the high potential clinical impact of PSMA PET on salvage radiotherapy planning [33]

The first excellent study was presented by Calais et al. [33]. This was a collaboration between the US and Germany. The authors reviewed 252 patients with early biochemical relapse after prostatectomy who had been scanned with ^68^Ga-PSMA-11 PET/CT to define the impact on RT planning (Fig. 10). ^68^Ga-PSMA-11 PET/CT affected salvage radiation therapy planning in 55% of patients with early biochemical recurrence (PSA <1 ng/ml) after radical prostatectomy.

Schwarzenboeck et al. [34] (Rostock, Germany) analysed the use of ^68^Ga-PSMA PET/CT in 38 patients with prostate cancer with a high risk or persistence or biochemical recurrence who were candidates for radiation therapy. In about 70% of these patients there was a clear direct impact on radiation therapy in terms of a change in the planning target volume and the addition of booster doses. Another interesting study was presented by Freitag et al. [35] (Munich, Germany) on the use of ^18^F-PSMA-1007 PET/MRI combined with integrated multiparametric PET/MRI for imaging prostate cancer. This promising high-resolution coregistered PET/MRI protocol was completed in 1 h and was found to be feasible for the patients, and also appeared to result in fewer artefacts in the prostatic fossa than ^68^Ga-PSMA 11 (due to low bladder accumulation of ^18^F-PSMA-1007).

Schmidkonz et al. [36] (Erlangen, Germany) presented clinical data on the use of ^99m^Tc-MIP-1404, a SPECT PSMA ligand, in prostate cancer patients. They enrolled 380 subjects and demonstrated high accuracy in detecting PSMA-positive lesions with a very good detection rate of 89% in patients with a PSA level ≥2 ng/ml and 40% in patients with a PSA level <2 ng/ml. Another example of outstanding work was presented by Haefliger et al. [37] (Lausanne, Switzerland). They compared the use of ^18^F-choline (FCH) and ^68^Ga-NODAGA-MJ9 (MJ9), a bombesin analogue and GRP receptor antagonist, for PET/CT imaging in 15 patients with histologically proven prostate cancer with a mean Gleason score (GS) of 7 ± 1 (6–9) and a mean PSA level of 40 ± 73 ng/ml. MJ9 uptake was significantly higher in the prostate and lymph nodes than FCH uptake, while FCH uptake was significantly higher than MJ9 uptake in bone lesions, confirming the role of bombesin analogues in the initial staging of prostate cancer patients.

Batra et al. [38] (New York, USA) reported a study of the use of ^89^Zr-df-IAB2M for PET/CT imaging of prostate cancer in a safe administration in nine men with a presurgery median PSA level of 8 ng/mL and a GS of ≥7 (range 6–9) in 89% of patients. IAB2M is an anti-PSMA minibody excreted via the hepatic route and in this phase 2a clinical trial showed excellent targeting of prostate cancer lesions and a very low urinary excretion in comparison with small-molecule PSMA ligands, with which bladder activity can compromise visualization in the prostatic bed.

## Radionuclide therapy and dosimetry

Radionuclide therapy is becoming an ever more important part of nuclear medicine practice with optimization of therapies, alpha therapies showing excellent clinical results, and new therapeutic approaches entering clinical trials.

A multicentre study presented by Sharma et al. [39] (Hershey, USA), that pooled the data from three randomized studies, evaluated the efficacy and safety of adding selective internal radiation therapy (SIRT) using ^90^Y resin microspheres to first-line mFOLFOX chemotherapy in 1,103 patients with liver metastases from colorectal cancer (mCRC). The addition of SIRT to first-line chemotherapy in patients with liver-only and liver-dominant mCRC led to an improvement in objective response rate (*p* = 0.001) and liver-specific progression-free survival (*p* < 0.001).

Dizdarevic et al. [40] (Brighton, UK) presented a multicentre study involving various centres in Europe and the USA called REASSURE, an observational study on ^223^Ra, a targeted alpha therapy, in which 1,106 patients with metastatic castration-resistant prostate cancer (mCRPC) were enrolled. The interim analysis included data from 583 patients who had completed chemotherapy. Patients not previously treated with chemotherapy had less advanced disease at baseline than those who had received chemotherapy, and side effects (drug-related serious adverse events and treatment-emergent drug-related adverse events, most frequently gastrointestinal or haematological) were more frequent in patients who had had prior chemotherapy, influencing the dropout rate of alpha therapy.

**Fig. 11:**
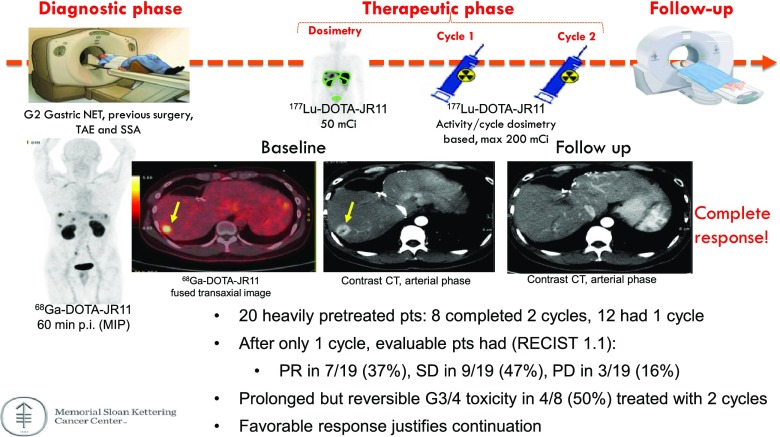
Theranostic trial of somatostatin antagonists ^68^Ga-OPS201 and ^177^Lu-OPS201 in well-differentiated neuroendocrine tumours (NET), revealing high safety and excellent response rates even after only one cycle of therapy with ^177^Lu-OPS201 [41]

Reidy et al. [41] (New York, USA) reported a phase I theranostic trial evaluating the safety and radiation dosimetry of the SSTR2 antagonist JR11 in 20 patients with metastatic well-differentiated NET. The preliminary data showed that all tumours were detected by ^68^Ga-OPS202 and therapy with ^177^Lu-OPS201 was associated with a very impressive response in more than 80% of patients, even after one cycle, indicating that this theranostic combination is highly promising for imaging and therapy (Fig. 11).

**Fig. 12:**
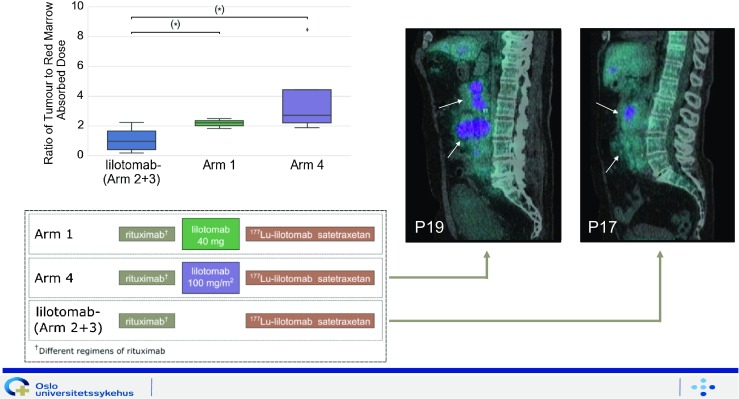
Results of early phase clinical trials with ^177^Lu-lilotomab satetraxetan targeting the CD37 antigen. Predosing with lilotomab prior to antibody–radionuclide conjugate therapy with ^177^Lu-lilotomab satetraxetan significantly increases the ratio of tumour to red marrow absorbed dose in patients with non-Hodgkin lymphoma [42]

Blakkisrud et al. [42] (Oslo, Norway) reported the results of early phase clinical trials of the use ^177^Lu-lilotomab satetraxetan (Fig. 12), a novel anti-CD37 antibody–radionuclide conjugate, for the treatment of non-Hodgkin lymphoma. Four different combinations of predosing and pretreatment were used. They enrolled 16 patients (pretreated with different regimens of rituximab) and there was a significantly higher red marrow dose in patients without predosing with anti-CD37 antibody radionuclide than in those with predosing (*p* = 0.04 and *p* = 0.05), indicating that predosing had a mitigating effect on red marrow absorbed dose, which would allow an increase in the tumour to red marrow absorbed dose ratio.

Scheidhauer et al. [43] (Munich, Germany) presented results of a pilot study in 12 patients evaluating the feasibility, safety and therapeutic efficacy of intravesical instillation of ^213^Bi-anti-EGFR antibody in reducing recurrent bladder cancer. All patients showed excellent tolerance of this treatment without any side effects. SPECT/ CT monitoring clearly revealed the location of the ^213^Bi-anti-EGFR antibody immunoconjugate in the bladder, and treatment resulted in a documented complete eradication of tumour cells in 25% of patients.

## EANM 2017 specials

This category was introduced in this year’s Highlights Lecture to point out some particularly memorable submissions and presentations that stress the speciality of nuclear medicine in various aspects. We selected the most unusual abstracts, one abstract with the highest clinical impact, to show that science in the end improves patient care, and the radiopharmaceutical of the year indicating the potential of nuclear medicine to expand into new fields in years to come.

A very unusual abstract, was submitted by Amor-Coarasa et al. [44] (New York, USA). They evaluated the use of a 3D printer to print a synthesis module for radiopharmaceutical preparations. By this method they established a complex multistep radiosynthesis including distillation that was sufficiently robust and reliable for routine clinical use, and thereby reduced costs considerably. Another unusual abstract was a “naturalistic study”. This was a multicentre study presented by Guedj et al. [45] (France) evaluating the use of florbetaben PET/CT for amyloid plaque imaging as compared with other approaches in the work-up of dementia patients. The authors demonstrated that the use of amyloid plaque imaging is superior to cerebrospinal fluid analysis, which is not only definitely more invasive and aggressive but is also less effective. Shiri et al. [46] presented a most unusual and scientifically highly interesting paper. These authors sought to predict lung metastases in patients with soft tissue sarcoma applying advanced machine learning to radiomic features. The unusual aspect, however, was the collaboration between Iranian universities and universities in the US, showing that science is above politics.

**Fig. 13:**
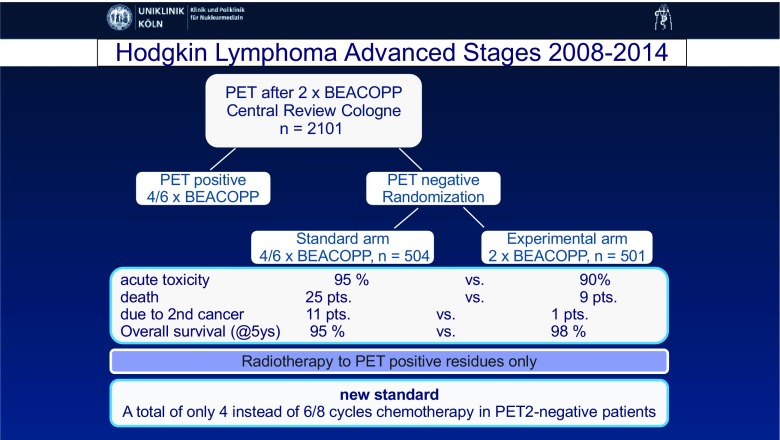
Treatment reduction in patients with advanced stage Hodgkin lymphoma and a negative interim PET scan. Final results of the international, randomized phase 3 HD18 trial by the German Hodgkin Study Group [47]

The abstract with the highest clinical impact was presented by Kobe et al. [47] (Cologne, Germany). This was a multicentre randomized double-blind study in which 2,101 patients with advanced stage Hodgkin lymphoma were enrolled. The aim of the study was to investigate the possibility of reducing the number of chemotherapy cycles in patients with a negative interim PET scan (Fig. 13). The study showed that the standard six or eight courses of eBEACOPP therapy can be reduced to only four courses in patients with a negative interim FDG PET scan without loss of lymphoma control. The improved tolerability of the de-escalated treatment strategy also resulted in a significant increase in overall survival (97.7%, *p* = 0.006)

**Fig. 14:**
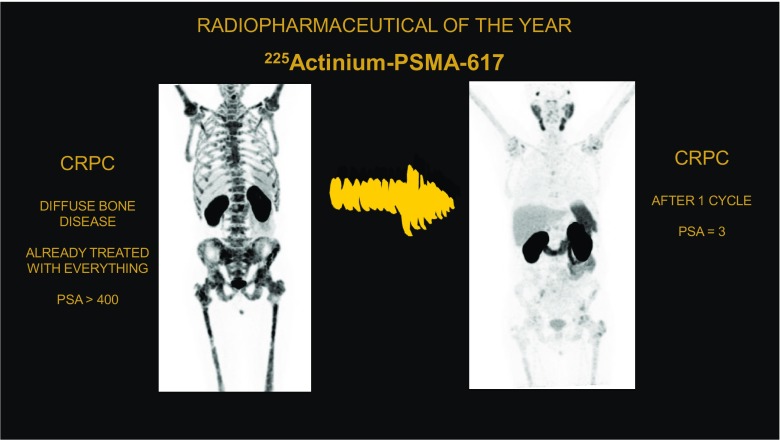
An impressive case of treatment response after only one cycle of ^225^Ac-PSMA 617, the radiopharmaceutical of the year at EANM 2017. Data on therapeutic efficacy [48]

^225^Ac-PSMA 617 was chosen for the special award of the radiopharmaceutical of the year. The data for this promising compound for alpha radiation therapy in advanced prostate cancer were presented by Kratochwil et al. [48] (Heidelberg, Germany). They enrolled patients with castration-resistant prostate cancer already treated with every available drug and, for comparison, patients treated with this novel alpha therapy. This new therapeutic radiation-approach showed favourable low haematological toxicity and also remarkable antitumour activity in terms of objective response and progression-free survival (Fig. 14).

## Conclusion

EANM 2017 again showed the high potential of nuclear medicine to be involved in the translation of basic science into clinical applications and in novel ways to diagnose and treat an ever greater number of diseases. This could only have been achieved by great contributions from all over the world.

We particularly mention the most active institutions, all of them submitting more than 20 abstracts to the meeting (summarized in Table 1), and to acknowledge the authors most active during EANM 2017: Peter Bartenstein from Munich, Gianmario Sambuceti from Genova, and Markus Schwaiger from Munich.

**Table 1:** Institutions submitting more than 20 abstracts to EANM 2017 (in alphabetical order according to the city of origin)

Centre	City	Country
Sant Orsola	Bologna	Italy
Universitätsklinik Essen	Essen	Germany
San Martino	Genova	Italy
University Medical Center (UMC)	Groningen	The Netherlands
Ludwig Maximilian University	Munich	Germany
Technische Universität	Munich	Germany
Radboud University	Nijmegen	The Netherlands
Uppsala University	Uppsala	Sweden
Medizinische Universität	Vienna	Austria

We asked whether our scientific community at EANM 2017 could “Make Nuclear Medicine Great Again”; we can definitely answer “Yes We Can”.
